# Recent progress in development of 2,3-diaminomaleonitrile (DAMN) based chemosensors for sensing of ionic and reactive oxygen species†

**DOI:** 10.1039/c9ra05298d

**Published:** 2019-09-27

**Authors:** Aruna, Bhawna Rani, Suman Swami, Arunava Agarwala, Debasis Behera, Rahul Shrivastava

**Affiliations:** Department of Chemistry, Manipal University Jaipur VPO-Dehmi-Kalan, Off Jaipur-Ajmer Express Way Jaipur Rajasthan India 303007 rahul.shrivastava@jaipur.manipal.edu arunava.agarwala@jaipur.manipal.edu

## Abstract

2,3-Diaminomaleonitrile (DAMN) has proved to be a valuable organic π-conjugated molecule having many applications in the area of chemosensors for sensing of ionic and neutral species because of its ability to act as a building block for well-defined molecular architectures and scaffolds for preorganised arrays of functionality. In this article, we discussed the utilization of 2,3-diaminomaleonitrile (DAMN) for the design and development of chemosensor molecules and their application in the area of metal ion, anion and reactive oxygen species sensing. Along with these, we present different examples of DAMN based chemosensors for multiple ion sensing. We also discuss the ion sensing mechanism and potential uses in other related areas of research.

## Abstract 2

2,3-Diamniomaleonitrile (DAMN) is valuable π-conjugated organic scaffold molecule for designing of efficient chemosensors for sensing of ionic and Reactive Oxygen Species (ROS).

## Graphical abstract

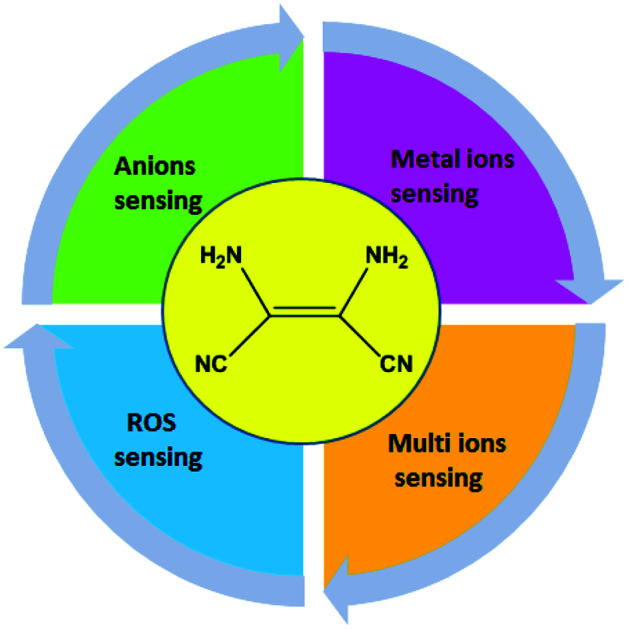

## Introduction

**Fig. 1:**
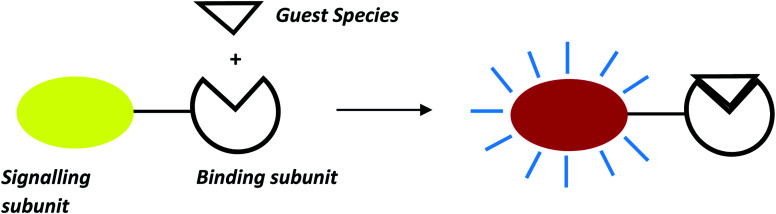
General approach for designing of chemosensor based on the signalling-binding subunits.

The design and development of chemosensor molecules mainly rely on the concepts of complementarity, co-cooperativity and preorganization.^1–3^ Sensor molecules derived from these concepts have a suitable orientation of functional groups which produce strong covalent and non-covalent interactions between sensors and target guest species.^4–6^ Different scaffolds like calixarenes, cyclotriveratrylene, cyclodextrin, boron-dipyrromethene (BODIPY), naphthalimides, crown-ethers/thia-crown-ethers, resorcinarenes *etc.* were successfully used for designing chemosensor molecules for sensing of specific ions and neutral species. These sensor molecules are developed in such a way that they can be functional in a competitive aqueous medium for wide applications in medical, biological and environmental domains.^7–9^ These chemosensor molecules exhibit specific photophysical properties like absorption of light at longer wavelength which facilitates naked eye detection of target species and interesting photoluminescence properties with high quantum yield.^10–17^ A general approach for development of optical chemosensors are coupling of signalling and binding sites either directly or through suitable spacer groups. For instance, binding unit binds guest species either through covalent or non-covalent interactions whereas signalling unit exhibits change in spectroscopic properties like colour, absorption maximum or fluorescence intensity upon guest interactions as shown in Fig. 1.^18^

Diaminomaleonitrile (DAMN) is a unique organic π-conjugated molecule in which electronic donor parts (–NH_2_ groups) and acceptor parts (–CN group) are linked by a single and double bond.^19–21^ The DAMN has tremendous potential to act as building block in designing of sensor molecules due to its fascinating electronic properties,^22,23^ which can be easily fine-tuned for sensible sensor design through functionalization by suitable functional group. In last few years, many excellent examples of DAMN based sensors have been published which proved its utility and versatility in research area of designing of molecular sensors. Reviews on different chemical reactions of DAMN as building block for synthesis of heterocyclic entities are previously reported in literature^24,25^ but to the best of our knowledge, reviews on DAMN based sensor molecules for sensing of ions and neutral guest species are still not available in literature. This review will focus on the recent development of DAMN based sensor molecules beginning with short discussion about the discovery and development of chemical structure of DAMN. Examples presented here are mainly grouped according to sensing properties. Finally, sensing mechanism and potential use in other related area of research will be discussed.

### Invention and chemical structure and properties of DAMN

**Fig. 2:**
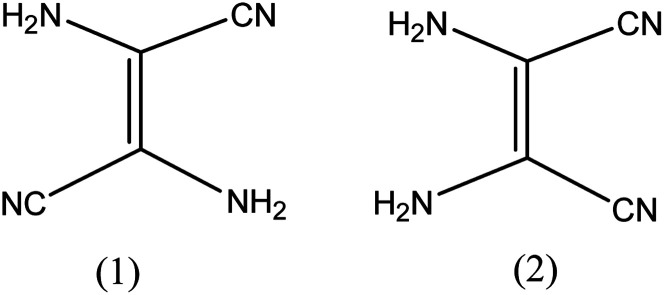
*Trans* and *cis* conformation of 2,3-diaminomaleonitrile.

The admittance of DAMN in the chemical world was perceived in 1873 by the efforts of Lange.^26^ He obtained a black crystalline compound directly from oligomerization of hydrogen cyanide. Thereafter Bedel *et al.* proposed that resulting black crystalline compound was the tetramer of HCN.^27^ Structure of diaminomaleonitrile had been in suspicion till 1954. In 1955–56, Webb^28^ and Bredereck^29^ suggested that the diaminomaleonitrile exists as tetramer. They also examined chemistry of tetramer with the help of infrared and ultraviolet absorption spectrum and concluded that DAMN exist as crystalline tetramer with absence of C–H bond.^30^ They carried out dipole moment measurements of DAMN in solution and concluded that molecule exits in *cis*-conformation (Fig. 2). The *cis* conformation of DAMN was confirmed by Bredereck from successful synthesis of mono- and di-acetyl derivatives of DAMN. Gryszkiewicz-Trochimowski obtained 2,3-dicyanopyrazine in more than 80% yield in mild reaction condition by condensation reaction between DAMN with glyoxal which again confirmed *cis* conformation of DAMN.^31^ Along with this, single crystal X-ray diffraction experiments unambiguously confirmed *cis* conformation of DAMN in crystalline state.^32^

### DAMN based chemosensors for anions

Anions are necessary for maintaining normal physiological functions of all living organisms due to the involvement of anions in almost every biological operation. Anions also play critical role in different industrial, chemical and environmental processes. Despite their significant role in different biological functions, higher or lower concentration of anions induce different deleterious effects on human health and environment.^33^ Due to deleterious effects of anions, demands of effective chemosensor molecules are drastically increased from last two decades. It was observed that selective sensing of anions with low detection limits are challenging area of research due to their wide range of geometries like spherical, trigonal, tetrahedral, helical and octahedral along with lower charge to radius ratio and tendency of anions to be protonated at low pH and in protic solvents make sensing of the anions even more difficult.

**Fig. 3:**
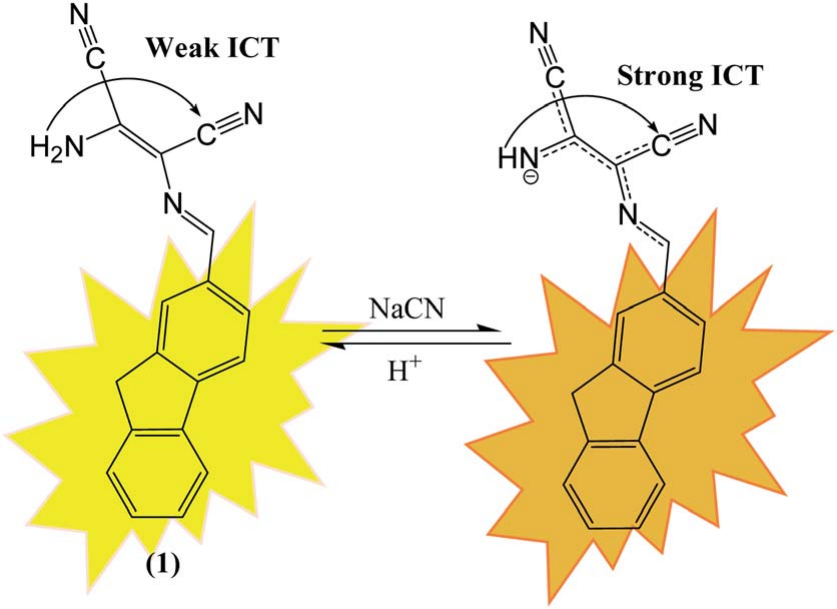
The mechanism for the fluorescence response of sensor (1) to CN^−^ ion.

**Fig. 4:**
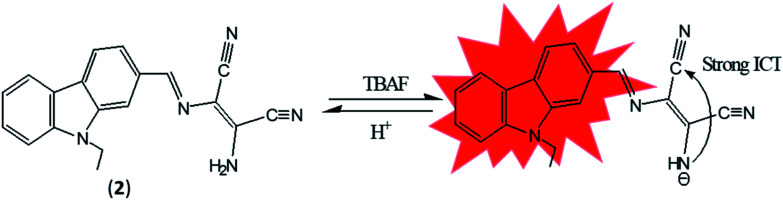
The sensing mechanism sensor (2) for F^−^ ion.

DAMN is proven as versatile scaffold for designing of sensor molecules for detection of anions due to its unique structural features. In this direction, two diaminomalenonitrile (DAMN) based colorimetric sensor molecules 2-(((*E*)-(9*H*-fluoren-2-yl)methylene)amino)-3-aminomaleonitrile (1) and (*E*)-2-(amino(((9-ethyl-9*H*-carbazol-2-yl)methylene)amino)methylene)maleonitrile (2) were developed in which DAMN was linked with fluorene and carbazole moiety respectively through imine linkage.^34^ Fluorene and its derivatives are electron rich fluorophore in which two benzene rings connected by a five-membered ring therefore provide extended π-conjugated orbitals. The fascinating electronic properties, high quantum yield and high sensitivity make fluorene an effective signalling unit for the development of chemosensors. The synthesized chemosensors (1 & 2) are π-conjugated organic molecules with electronic donor (NH_2_) and acceptor (–CN) unit linked by single and double bond leads significant electronics properties due to an intramolecular charge transfer (ICT). The synthesized sensor molecules displayed selective sensing of F^−^ and CN^−^ ions in presence of other anions through colorimetric changes and fluorescence responses (Fig. 3 and 4). The sensor (1) exhibited emission peak at 460 nm upon excitation at 380 nm. However, presence of CN^−^ ions in solution of sensor (1) shifted the emission peak to 480 nm. The observed 20 nm red shift was explained by enhancement of intramolecular charge transfer from –NH^−^ group to nitrile group, facilitated by hydrogen bond interaction of CN^−^ ions with amino group (Fig. 3). Other synthesized sensor molecule (2) exhibited peak at 400 nm in UV-visible spectrum which assigned for the charge transfer from NH_2_ to CN^−^ of diaminomaleonitrile (DAMN) (Fig. 4). Addition of fluoride ion to sensor (2) solution induced shift of absorption peak from 400 nm to 470 nm along with appearance of instance colour of reddish green. The sensing mechanism was further confirmed the by ^1^H NMR studies and DFT calculations.

**Fig. 5:**
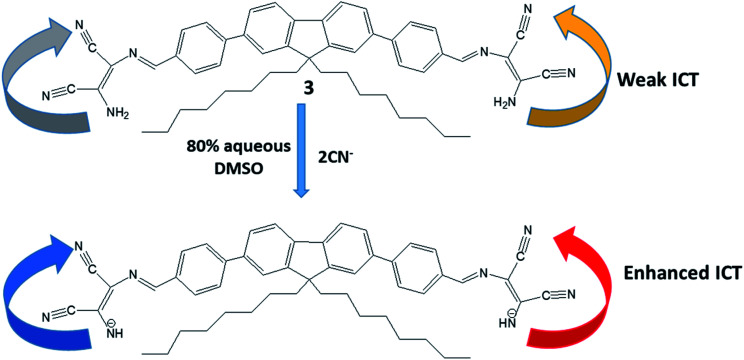
The mechanism for the fluorescence response of sensor (3) to CN^−^ ion.

In this direction, Son *et al.* have developed extended π-conjugated system (3) in which both ends of dioctyl fluorene–dibenzene were linked with two DAMN units through imine linkage (Fig. 5).^35^ The incorporation of two DAMN made this sensor (3) very efficient for detection of CN^−^ ion in aqueous medium in nano-molar concentration which is much lower than the concentration level recommended by World Health Organization guidelines. The sensor (3) exhibited intense colour change from yellow to red along with fluorescence turn-on response upon interaction with CN^−^ ion. Additionally, it was observed that in presence of CN^−^ ion, absorption peak at 406 nm in UV-visible spectrum was shifted to 478 nm with isosbestic point at 446 nm. The shift in absorption maxima was explained by the deprotonation of NH_2_ of DAMN coupled with intermolecular charge transfer led to enhancement of donor-to-acceptor ICT transition (Fig. 5). They also examined the sensitivity of sensor (3) towards other anions such as H_2_PO_4_^−^, NO_3_^−^, AcO^−^, F^−^, Cl^−^, Br^−^ and I^−^ but none of these ions showed any responses in absorption, emission spectrum and colorimetric sensing.

**Fig. 6:**
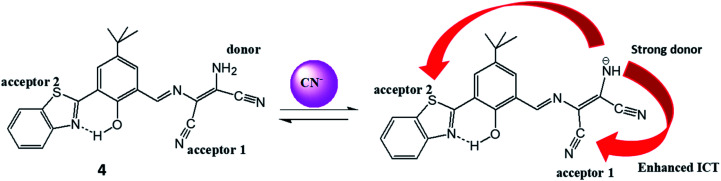
The mechanism for the fluorescence response of sensor (4) to CN^−^ ion.

Another DAMN derived biocompatible duel channel probe (4) was synthesized from hydroxyl benzothiazole moiety and 2,3-diaminomaleonitrile for selective detection of toxic CN^−^ ions in 50% aqueous DMF.^36^ The merits of the probe (4) were; (i) reversibility; (ii) non-cytotoxicity; (iii) excellent cell viability; (iv) 0.16 μM detection limit and (v) imaging of CN^−^ in live fibroblast L929 cells. The interaction of CN^−^ ion with probe (4) resulted in disappearance of absorption peak at 370 mm with evolution of 445 nm peak with isosbestic point at 390 nm in UV-visible spectrum. Along with this, colorimetric change from yellow to red was observed upon addition of CN^−^ ion into solution of probe (4). These observations were explained by the electronic push–pull effect from amino group of DAMN to nitrile groups resulted from interaction of anion with N–H bond which led polarization of N–H bond and then deprotonation process. In fluorescence studies, probe (4) exhibited weak emission peak at 517 nm upon excitation at 450 nm which was explained by the fact that photo-induced electron transfer process generated from amino group suppressed the emission response. The observed weak emission peak at 517 nm was significantly intensified in response to CN^−^ ion probably due to the abstraction of proton of amino group by CN^−^ ion which resulted strong intramolecular charge transfer process (ICT) from amine to nitrile units of DAMN. The strong ICT prevent photo-induced electron transfer process resulted in significant fluorescence enhancement (Fig. 6). Additionally, fluorescence turn-on behaviour of probe (4) upon interaction with CN^−^ ion can also supported from suppression of ESIPT process (excited state intramolecular proton transfer) because of deprotonation of benzothiazole–phenol unit.

Indole and its derivative are interesting heterocyclic scaffolds attracted immense interest of medicinal and organic chemists due to its important application in medicinal and biological field. The structure of indole comprises with pyrrole and benzene rings makes it attractive fluorophore for designing of sensible sensor molecules. Additionally, the presence of N–H group in indole moiety can be utilized for sensing of anions through hydrogen bond interaction. To utilize these properties, a duel channel chemosensor (5) comprising two indole moieties was synthesized from condensation reaction of diaminomaleonitrile with indole-3-carboxaldehyde for sensing of fluoride ion.^37^ Reported sensor 5 selectively sensed fluoride ion without interferences of other anions. The significant changes in emission and absorption spectrum upon addition of fluoride was explained on the basis of hydrogen bond interaction between F^−^ ion and N–H group of indole moiety. The sensing studies of chemosensor (5) has been validated by ^1^H NMR titration and spectrophotometric titrations.

Apart from DAMN based organic molecules, DAMN based metal complexes also showed interesting sensing properties. Two such complexes uranyl-*N*,*N*′-bis(5-*tert*-butyl-2-hydroxybenzylidene)-1,2-dicyano-1,2-ethenediamine (6) and uranyl-*N*,*N*′-bis(3,5-ditertbutyl-2-hydroxybenzylidene)-1,2-dicyano-1,2-ethenediamine (7) were developed by Cort and his group by reaction of salycilaldehydes derivatives, 1,2-diaminomaleonitrile and uranyl acetate for selective sensing of halide ion in chloroform and dichloromethane.^38^ Sensor 6 and 7 showed selectivity trends for halide ions in order of F^−^ > Cl^−^ > Br^−^ which is in good agreement of hardness of anions. Among 6 and 7, compound 7 showed change in colour upon addition of fluoride and chloride ion but it exhibits fluorescence “turn-on” response only with fluoride ion in both CH_2_Cl_2_ and CHCl_3_. The weak sensing response of 6 towards halide ion was explained from its higher stability of the dimer formation as compare to 7. Further, they calculated distances of U⋯X (X = F^−^, Cl^−^, Br^−^) from DFT calculation for providing support to metal center and bound halide ions interactions.
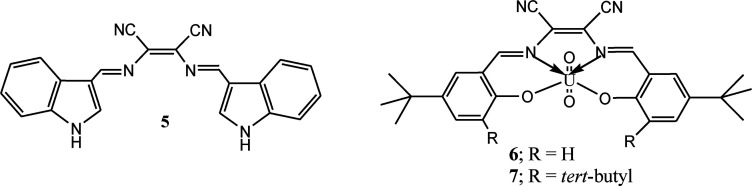


### DAMN based chemosensors for metal ions

Metal ions are essential for many environmental, industrial, biological and chemical processes. Some metal ions like Cu^2+^, Zn^2+^, Fe^2+^/Fe^3+^, Co^2+^, Mn^2+^*etc.* are necessary to perform numerous biological functions and enzymatic actions to support life cycles. However high dosages, long-lasting exposure, imbalance in regulations of these ions lead different adverse effect on human health and environment.^39^ For instance, excess accumulation of Cu^2+^ ion causes kidney damage and Wilson disease, excess intake of Mn^2+^ lead to manganism and excess Zn^2+^ intake causes vomiting, headache and nausea. On the other hand, some metal ions like Hg^2+^, Cd^2+^, Pb^2+^, Cr^3+^ and As^3+^ are toxic in nature without any beneficial effects on human health. Literature revealed that accumulation of these ions lead serious illnesses like damages of central nervous system, brain dysfunction, eyes, lungs, mental retardation and many more. The direct or indirect involvement of metal ions in serious diseases increases demands of effective organic molecules based sensing systems for quantitative and qualitative sensing of metal ions at low detection level therefore various research groups are involved in developing such systems for easy and selective detection of metal ions. DAMN derived sensor molecules for metal ions have been proven to be an attractive choice due to inimitable structural features which arise fascinating electronic properties.

To develop sensor molecules for metal ions, aza-crown ether moieties have been introduced into DAMN scaffold, as aza-crown ethers have excellent capability for binding to specific metal ions with stable complexation because of the nitrogen/oxygen are situated at interior of the ring to coordinate with target cation. The selectivity of these molecules depends on size of cavity as well as number of oxygen and nitrogen atoms. Thus, Zhou and his group introduced aza-crown ether moiety into diaminomaleonitrile through condensation reaction between diaminomaleonitrile with 4-(1,4,7,10-tetraoxa-13-aza cyclopentadecyl)benzaldehyde or 4-(1,4,7,10,13-pentaoxa-16-aza cycloctadecyl)benzaldehyde in benzene with aim to combine two binding sites in single sensor molecules.^40^ The sensor molecules (8 & 9) having different cavity size of aza-crown ether moieties were examined for sensing of metal ions. It was found that sensor 8 having smaller cavity size as compare to 9 showed different colorimetric responses for Hg^2+^ and Cu^2+^ ions. For example, 8–Hg^2+^ turned to pale yellow whereas 8–Cu^2+^ turned to colorless within a wide pH range of 5.5–10.5. In UV-visible spectrum, sensor 8 showed absorption peak at 420 nm which was decreased and appearance of a peak at 336 nm with isosbestic point at 366 nm upon stepwise addition of Cu^2+^ ion. The mole fraction at 0.5 in Job's plot revealed 1 : 1 binding stoichiometry between Cu^2+^ and 8 and stability constant was found to be 1.04 × 10^4^ M^−1^. In case of sensor 9 with larger cavity as compare to 8 showed different sensing behavior for Cu^2+^ ion. They found that upon addition of Cu^2+^ to 9, absorptions peaks centered at 300 and 420 nm were decreased with isosbestic points at 316 nm and 373 nm while addition of more than 0.8 equivalents of Cu^2+^ ion, new absorption peak at 260 nm appeared with new isosbestic point at 297 nm. It was also observed that sensor 9 showed much lower detection limit for Cu^2+^ ion in comparison of 8. These differences in selectivity for Cu^2+^ ion was observed due to consequences of difference in cavity size of aza-crown ethers. The sensitivity of 8 was explained by the fact that Cu^2+^ ion coordination with the donor nitrogen atom of aniline group of DAMN while larger cavity of aza-crown ether and DAMN in 9 have competition in sensing of Cu^2+^ ion which was further supported by theoretical calculations.
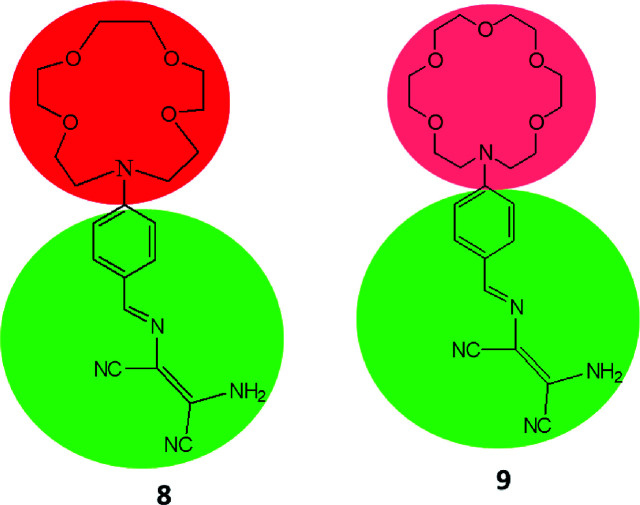


**Fig. 7:**
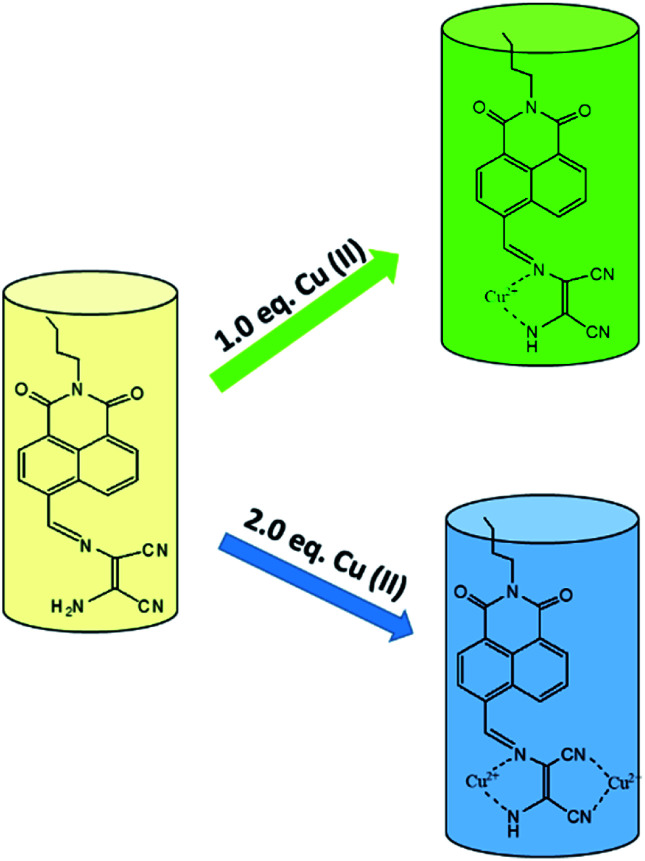
Colorimetric responses sensor (10) with different equivalent of to Cu^2+^ ion.

Most of chemosensor molecules showed fluorescence turn-off responses for sensing of Cu^2+^ ion due to the paramagnetic nature of Cu^2+^ ion which may sometime reduce the sensitivity and selectivity of sensor molecules. In comparison to this, ratiometric and turn-on fluorescent sensors are much better options for sensing of Cu^2+^ ion due to better sensitivity and selectivity. In this context, DAMN based turn-on fluorescent sensor for Cu^2+^ ion using naphthalimide moiety was reported.^41^ Naphthalimide moiety is exceptional fluorogenic in nature widely used in designing of chemosensor molecules. The emission and absorption properties of naphthalimide derivatives largely depend upon substituents on naphthalimide skeleton and can be fine-tuned as per need. The reported DAMN conjugated naphthalimide sensor (10) showed two different emission channel upon addition of different concentrations of Cu^2+^ ions (Fig. 7). For instance, addition of less than 1.0 equivalent of Cu^2+^ ion, both absorbances at 325 and 405 nm decreased while addition of Cu^2+^ ion beyond 1.0 eq. induced increase of the absorption peak at 325 nm and a monotonic decrease at 405 nm. In fluorescence titration experiments, lower than 1.0 equivalent of Cu^2+^ ion, the intensity of emission peak at 522 nm was increased while further addition of Cu^2+^ ion, peak at 522 nm disappeared and a new emission peak gradually appeared at 425 nm. In the sensing process the first equivalent of Cu^2+^ ion coordinate with nitrogen atoms of imine and amine group and block the photo-induced electron transfer which results in significant fluorescence enhancement at 522 nm. Further, binding of second equivalent of Cu^2+^ ion with nitrile group of DAMN enhance the electron-withdrawing ability of imine group which results shift of emission peak from 522 to 425 nm. These observations were obtained only in acetonitrile solvent system whereas in other solvents like DMF, THF, water and DMSO only fluorescence enhancement at 522 nm was observed which clearly indicated that binding ability of nitrile group to Cu^2+^ was weak in nature and was replaced by the solvents having strong binding ability. In the continuation of this work, an interesting DAMN based fluorescent turn-on sensor for Cu^2+^ ion using indoline moiety (11) was reported with excellent detection limit of 6.18 × 10^−8^ mol L^−1^.^42^ The synthesized sensor 11 displayed large Stokes shift of 146 nm which can reduce the impact of automatic fluorescence and self-extinguishing thus suitable for wide applications. The fluorescence turn-on response of 11 was described by prevention of the photo-induced electron transfer (PET) process from indole moiety (donor) to DAMN unit (receptor).
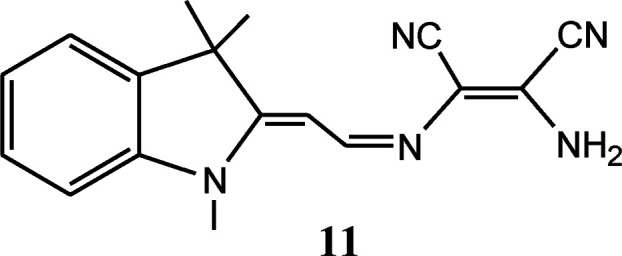


**Fig. 8:**
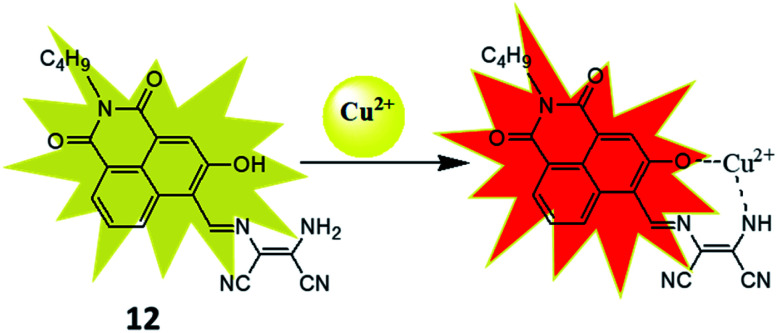
The sensing mechanism of sensor (12) for Cu^2+^ ion.

To improve the sensitivity of sensor, 3-hydroxy-naphthalimide was used to construct effective naphthalimide based sensor molecules in which oxygen atom of hydroxyl group also participate in binding process.^42^ The resulting sensor (12) was examined for sensing of different metal ions and result showed selective colorimetric response from yellow to pink only for Cu^2+^ ion with detection limit of 1.6 μM. For instance, 12 showed strong absorbance at 457 nm which was decreased on addition of Cu^2+^ ion whereas new peak at 565 nm was evolved and enhancement of absorbance at 565 nm was more than 50 fold. The selectivity of the receptor was also checked by mixing other interfering metal ions with Cu^2+^ ion, even in the complex mixture sensor 12 selectively detected copper ion. The nature of the interaction between the 12 and Cu^2+^ ion was established through various analysis. The Job's plot indicated the formation 1 : 1 complex and DynaFit curve fitting program also indicated formation of 1 : 1 complex with association constant (*K*_asso_) 5.9 × 10^4^ M^−1^. An *m*/*z* peak at 449.3 in mass spectra indicated formation of a 12–Cu^2+^ complex in which Cu^2+^ ion binded with oxygen atom of hydroxyl group and nitrogen atom of amino group (Fig. 8). The use of the receptor in practical application was demonstrated on simulated semiconductor wastewater by extracting the Cu^2+^ ion in DMSO–ethyl acetate (containing the receptor) medium at 4.8 pH. After extraction the image of the ethyl acetate phase was captured using a smartphone and the image was analysed using RGB Grabber (Shunamicode) application in smartphone. A calibration curve made by red/green ratio as function of Cu^2+^ ion gave satisfactory Cu^2+^ concentration result in simulated semiconductor wastewater.

**Fig. 9:**
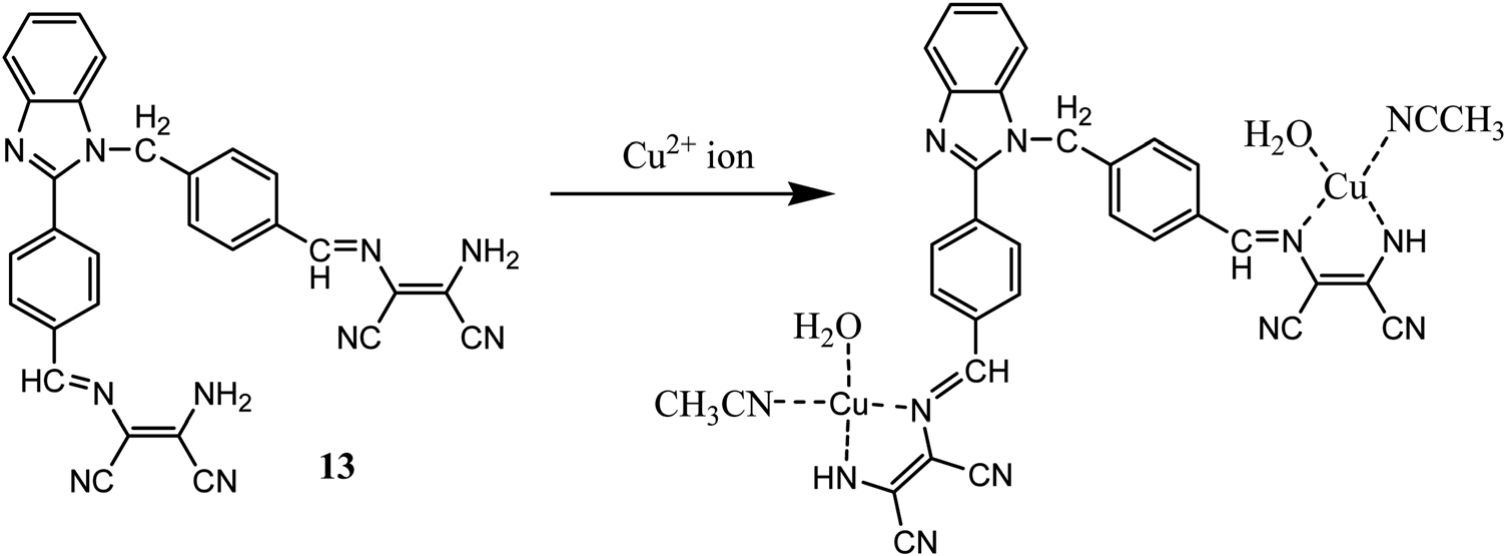
The sensing mechanism of sensor (13) for Cu^2+^ ion.

In another report benzimidazole derivative was coupled with two molecules of DAMN as optical properties of benzimidazole is highly sensitives towards metal ions interaction even in micro to nano level concentration.^43^ Sensor 13 demonstrated selective turn on fluorescent sensing behaviour for Cu^2+^ ion with 0.49 μM detection limit which was much lower concentration than permissible limit of Cu^2+^ ion in drinking water allowed by United States Environmental Protection Agency.^44^ The emission spectrum of 13 showed weak emission peak at 405 nm which enhanced to 155-fold with large Stokes shift of 60 nm on addition of Cu^2+^ ion. The most important aspect of sensor 13 is that it can selectively detect Cu^2+^ ion in presence other interfering ions in aqueous medium in wide pH range. The binding mode of sensor with Cu^2+^ ion was determined using Job's plot, mass-spectrometric methods as well as DFT calculation. All these methods indicated 1 : 2 stoichiometry ratio of 13–Cu^2+^ complex (Fig. 9). The *m*/*z* peak at 752.5849 in mass spectrometric analysis further supported proposed 1 : 2 13–Cu^2+^ ion complex. DFT analysis showed HOMO (−6.54 eV) and LUMO (−3.01 eV) band gap as 3.53 eV, whereas complexation with Cu^2+^ ion reduced the band gap to 3.24 eV. The fluorescence images of cell (HepG2) was carried out in presence of the 13, indicated the permeability and detection ability of the sensor in live cells.

Coumarin derivatives have high fluorescence quantum yield, large stoke shift and high photo-stability thus extensively utilized in designing of fluorescent sensor molecules. A coumarin–DAMN based fluorescent sensor with longer emission wave length was developed in which 7-diethylamino group (electron donor) and DAMN (strong electron acceptor) were introduced into coumarin scaffold to generate an extensive donor–acceptor system which enhanced ICT process.^45^ Although, synthesized sensor (14) exhibited weak fluorescence due to the quenching effect of nitrile group as well as its flexible structure, the intensity of emission peak at 620 nm was significantly increased up to 35-fold upon interaction with Zn^2+^ ion along with blue shift of 50 nm. The enhancement of fluorescence intensity was observed due to reduced ICT process and inhibition of conformational change upon combination with Zn^2+^ ion. The Job's plot revealed the 1 : 1 binding between 14 and Zn^2+^ ion which was supported by mass spectrometry. In addition, sensor (14) was used for Zn^2+^ ion imaging in HepG2 cells over Cd^2+^ ion which usually responses together with Zn^2+^ ion.
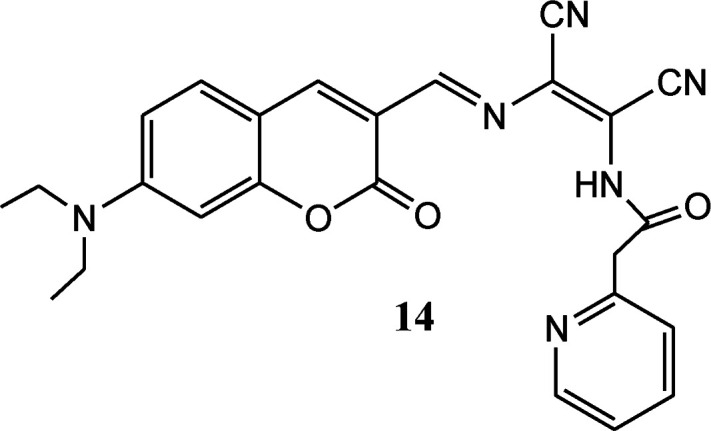


In continuation of our research efforts on developing sensor molecules,^46–51^ we synthesized hybrid chemosensors by combining diaminomaleonitrile, 4-formyl-1-substituted phenyl-pyrazole-3-carboxylate and 2-hydroxy-1-naphthaldehyde molecules.^52^ These three moieties were joined together by imine linkages and were capable of sensing Mn^2+^ and Zn^2+^ ion in complex mixture through colorimetric and fluorometric response. On addition Mn^2+^ and Zn^2+^ salt solutions the pale yellow coloured solution of the sensor (15–17) was immediately changed to dark brown-red and bright yellow colour respectively. The evaluation of interference by different metal ions like Al^3+^, Cr^3+^, Cu^2+^, Fe^3+^, Hg^2+^, Co^2+^, Pb^2+^, Cd^2+^ and Ni^2+^ indicated the sensor can selectively and efficiently detect Mn^2+^ and Zn^2+^ ions in mixture. The immediate colorimetric response made the molecule for possible colorimetric probe for Mn^2+^ or Zn^2+^ ions. The UV-vis spectroscopic study of the probe molecule showed three absorptions peaks at 328, 406 and 426 nm with highest absorbance (*λ*_max_) at 406 nm. On gradual addition of Mn^2+^ ion solution to probe molecule the 406 nm peak was completely disappeared and evolution of a new peak at 576 nm was observed. The evolution of new peak was complete with addition of 1.0 equivalent Mn^2+^ ion. Whereas the gradual addition of Zn^2+^ ion solution to probe solution resulted in formation of a new peak at 462 nm and the formation was complete with addition of 2 equivalents of Zn^2+^ ion. The Job's method and Benesi–Hildebrand plots indicated the 1 : 1 and 1 : 2 binding modes for Mn^2+^ and Zn^2+^ respectively. The binding constants for Mn^2+^ and Zn^2+^ were found to be 1.22 × 10^4^ M^−1^ and 3.31 × 10^8^ M^−2^ respectively. The practical applicability of the sensor was tested on tap water samples spiked with standard Zn^2+^ and Mn^2+^ ions and the results clearly indicated that the sensor can detect the presence of these ions even from aqueous medium. The fluorescent investigation on the sensor showed an emission peak at 507 nm upon excitation at 406 nm and the emission peak intensity decreased by 90% with addition of Mn^2+^ ion whereas the intensity of the 507 nm peak increased by 50% on addition of Zn^2+^ ion. The quenching of fluorescent intensity is due to binding of Mn^2+^ ion with the naphthalenic OH group and the imine nitrogen moiety, which causes internal charge transfer (ICT). The Stern–Volmer equation showed linear relationship with the quencher (Mn^2+^ ion) concentration, indicating quenching by the Mn^2+^ ion only. All these studies indicated potential application for detection for detecting Zn^2+^ and Mn^2+^ ions in water samples and which can become alternative to expensive techniques like AAS.
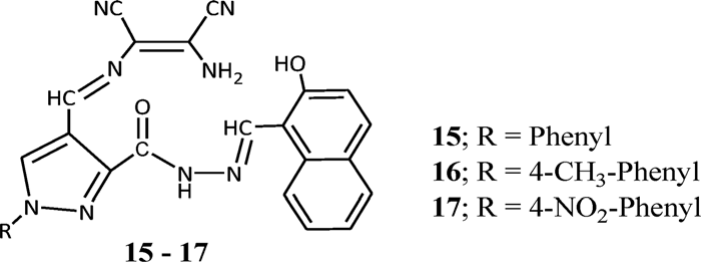


**Fig. 10:**
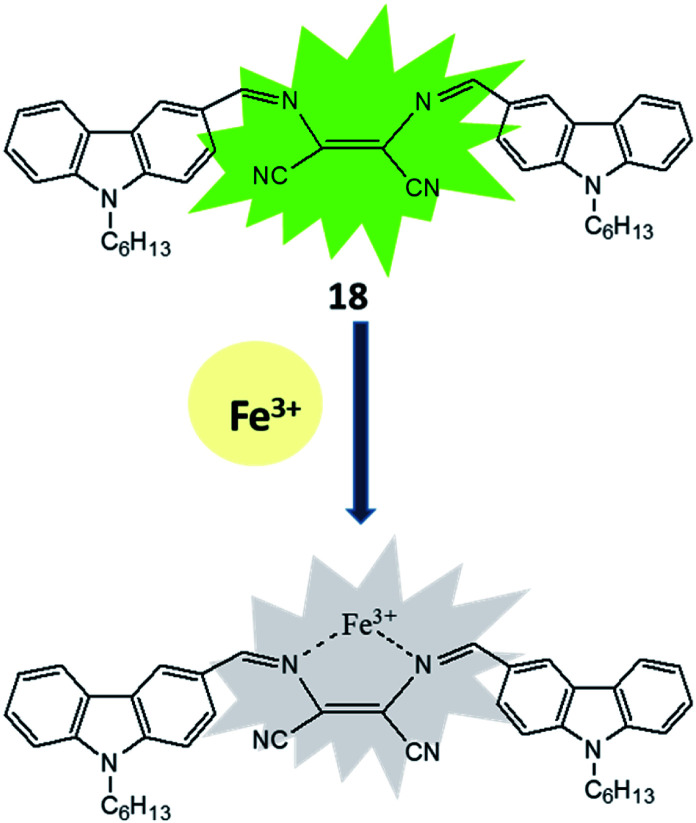
The sensing mechanism for sensor (18) or Fe^3+^ ion.

The existence, location and concentration of Fe^3+^ ions are extremely vital for execution of numerous biological functions in living organism whereas excess and deficiency lead various biological disorders in human body. In view of the paradox of Fe^3+^ ion, carbazole-diaminomaleonitrile derived fluorescence turn-off sensor (18) was developed for Fe^3+^ ion in which carbazole moiety was used a signalling unit.^53^ A strong fluorescence emission peak was appeared between 380–420 nm under excitation at 280 nm due to highly conjugated structure of sensor in which two carbazole unit connected on both end of DAMN through imine bond. The addition of Fe^3+^ ion in the solution of sensor induced significant fluorescence quenching while other metal ions like K^+^, Mn^2+^, Ag^+^, Co^2+^, Zn^2+^, Ni^2+^, Pb^2+^, Cd^2+^, Mg^2+^, Sr^2+^, Ca^2+^, Hg^2+^, Ba^2+^, Cu^2+^, Fe^2+^, Cr^3+^ and Al^3+^ did not influence the emission intensity of sensor. The nitrogen atoms of 18 were used for coordinating Fe^3+^ ion which induced internal charge transfer process from nitrogen of carbazole unit to Fe^3+^ ion causes significant fluorescence quenching (Fig. 10). Additionally, 18 showed fast response time with detection limit of 3.75 × 10^−8^ M for Fe^3+^ ion which make it useful for real time applications.

**Fig. 11:**
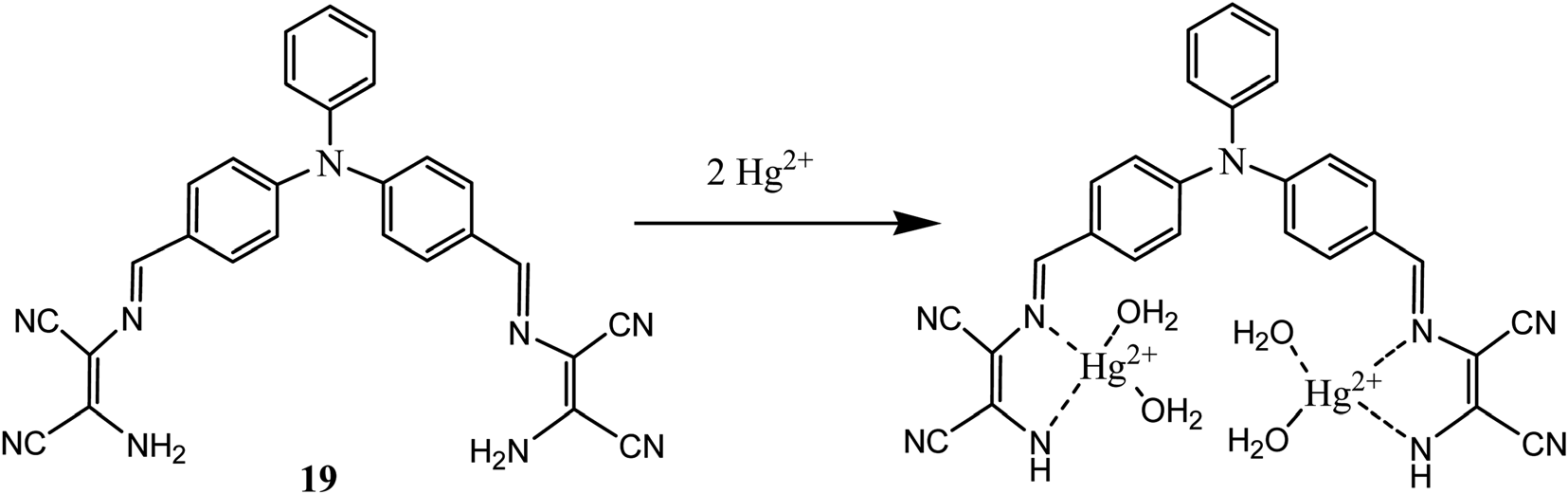
The 1 : 2 binding complexation between sensor (19) and Hg^2+^ ions.

Mercury exists in nature mainly in organic, inorganic and elemental forms. All these forms have strong affinity toward carboxyl, thiol and phosphate functional groups present in human body which causes serious toxic effects. DAMN moiety was also used in development of sensor molecules for sensing of mercury. In this context, a highly efficient reversible chromo-fluorescent molecular probe (19) was synthesized by conjugating triphenyl amine and DAMN where triphenyl amine act as signalling unit and DAMN act as a binding unit.^54^ The synthesized receptors (19) exhibited absorption peak centred at 447 nm which shifted to 508 nm upon addition of Hg^2+^ with appearance of instant orange colour from light yellow in acetonitrile : water (1 : 1) solution. The observed red shift (60 nm) in the absorption spectra is probably due to the enhancement of intermolecular charge transfer after complexation of the Hg^2+^ ion with receptor. In addition to this Job's continuous variation method indicated 1 : 2 binding stoichiometry between sensor (19) and Hg^2+^ ions (Fig. 11). The detection limit was found to be 5.2 μM at neutral pH in 50% aqueous acetonitrile solution.

**Fig. 12:**
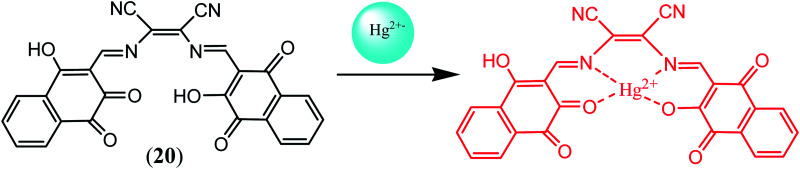
The binding mechanism between sensor (20) and Hg^2+^ ions.

Sekar *et al.* fabricated colorimetric test strips based on DAMN derived sensor (20) for convenient and efficient sensing of Hg^2+^ ions. These test strips showed visible color changes for sensing of Hg^2+^ ion in acetonitrile. The chemosensor (20) used in test strips was synthesized by the combination of diaminomaleonitrile and 3-hydroxy-1,4-dioxo-1,4-dihydronaphthalene-2-carbaldehyde.^55^ The colorimetric sensing abilities of sensor (20) for Hg^2+^ ion was again evaluated by UV-visible spectroscopy. It was observed that absorption bands of 20 at 335 nm and 481 nm were disappeared and new absorption bands at 421 nm and 595 nm were appeared on addition of Hg^2+^ ion. It was found that absorption peak at 595 nm reached its saturation point upon addition of 1.0 equivalent of Hg^2+^ ion indicating the formation of 1 : 1 binding stoichiometry. The Job's plot, ESI-mass spectrometry analysis ^1^H NMR titration and DFT calculations further confirmed the 1 : 1 binding mode [Fig. 12].

Huo *et al.* reported five diaminomaleonitrile based Schiff base compounds (21–25). These Schiff bases were prepared by condensation of substituted-benzaldehyde (electron donor) and diaminomaleonitrile (electron acceptor). These compounds showed selective and efficient sensing of Hg^2+^ ion in ethanolic aqueous medium with micro molar detection limit.^56^ These sensor molecules exhibited visual colour changes upon interaction with Hg^2+^ ion over other tested metal ions (K^+^, Ca^2+^, Ba^2+^, Co^2+^, Cd^2+^, Mg^2+^, Ag^+^, Na^+^, Fe^3+^, Cu^2+^, Ni^2+^, Al^3+^, Zn^2+^, Cr^3+^ and Pd^2+^) in aqueous medium. For example, addition of 1.0 equivalent of Hg^2+^ ion changed the colourless solutions of 21–24 to yellow whereas pale yellow colour solution of 25 changed to orange red.

**Fig. 13:**
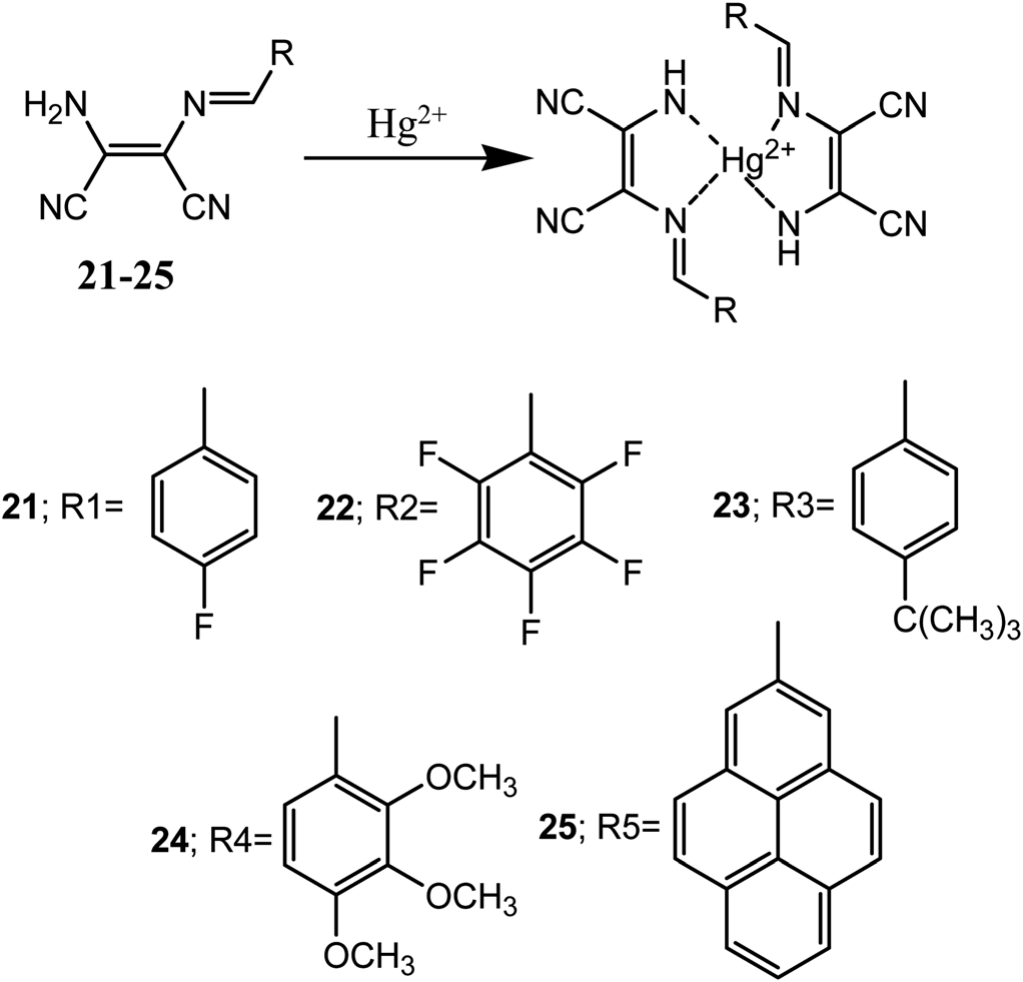
General 2 : 1 binding mode between sensor (21–25) and Hg^2+^ ions.

The sensor molecules (21–25) showed maximum absorbance peaks (*λ*_max_) centred at 362, 364, 365, 377 and 421 nm respectively. Addition of Hg^2+^ ion to solution of 24 caused a 28 nm red-shift (from 377 to 405 nm) and almost similar shifts in absorption peaks were observed with other sensor molecules (21–23 & 25). The binding mode of the sensors and Hg^2+^ ion was determined by Jos's plot method, indicated 2 : 1 binding stoichiometry which were confirmed by Benesi–Hildebrand equations (Fig. 13). The binding constants (*K*_asso_) was found in the range of 10^2^ to 10^3^ M^−1^. Crystals of sensor molecules (23 and 24) and its complex with Hg^2+^ were grown and single crystal X-ray diffraction study revealed that 23 crystallized in the monoclinic crystal system with *P*2_1_/*c* space group and the 24–Hg^2+^ complex crystallized in the monoclinic crystal system with *C*_2_/*c* space group.

Salen type ligands and its metal complexes have attracted immense attention in various fields like organic light-emitting diodes, catalysts, magnetic materials, supramolecular materials and cell imaging. These ligands and their complexes are easy to synthesis, reasonably stable and rich in photophysical properties. Furthermore, luminescent Salen ligands having π-conjugated tetradentate [O^N^N^O] chelating system are capable to coordinate with different metal ions, thus widely used in detection of wide-range of metal ions. Xiang group has recently prepared two Salen type ligands (26 and 27) by condensation reaction of diaminomaleonitrile and 4-(diethylamino)salicylaldehyde.^57^ Due to presence of diaminomaleonitrile moiety, it was possible to synthesize both *cis* and *trans* isomers. These isomers have pH sensitive groups such as hydroxyl, dimethylamine and –C=N and ease of acid catalysed hydrolysis of these two ligands were exploited to get wide range pH probe using these compounds. These Salen pH probes were able to detect pH ranging from 1–12 in both colorimetric and fluorometric modes. It is important to note that addition of OH^−^ ions (higher pH) the phenolic –OH group gets deprotonated and colour of the probe changes from pink to blue whereas addition of H^+^ ions (lower pH), colour of the probe solution changes from pink to purple due to protonation of imine nitrogen. Further addition of acid, probe gets hydrolysed to constituent diaminomaleonitrile and 4-(diethylamino)salicylaldehyde with disappearance of colour. All these protonated and deprotonated species were confirmed by ^1^H NMR measurements in which OH signal (*δ* = 11.71 ppm) disappeared upon addition of base and N=CH signal shifted from *δ* = 8.43 ppm to 9.60 with addition of acid.
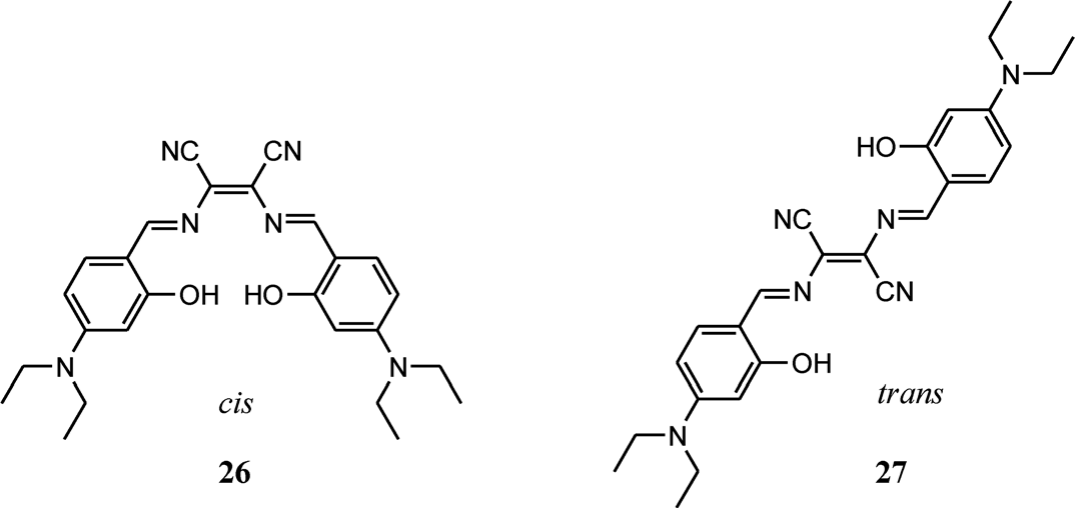


Among these two isomers *cis* isomer can act as tetradentate ligand whereas the *trans* form can act as bidentate ligand. It was observed that *cis* isomer can bind with various metal ions like Cu^2+^, Co^2+^ and Fe^3+^ and noticeable change in colour was observed by naked eye and fluorescence quenching response. In contrast to the *cis* isomer the *trans* isomer was able to detect Cu^2+^ ion selectively in ppb level by quenching the fluorescent intensity at 610 nm by 96% upon addition of 1.0 equivalent of Cu^2+^ ion and 100% quenching upon addition of 4.0 equivalents of Cu^2+^ ions. The investigation of Cu^2+^ ion detection mechanism indicated formation of a 1 : 1 *trans* ligand–Cu^2+^ complex, which then hydrolysed to diaminomaleonitrile and 4-(diethylamino)salicylaldehyde. The practical applicability of the *trans* isomer was checked by making the paper strip with the ligand and the dipping the strips in aqueous Cu^2+^ ion solution, acid and base solution. The obvious colour changes were observed as in solution.

### DAMN based chemosensors for multiple ions

**Fig. 14:**
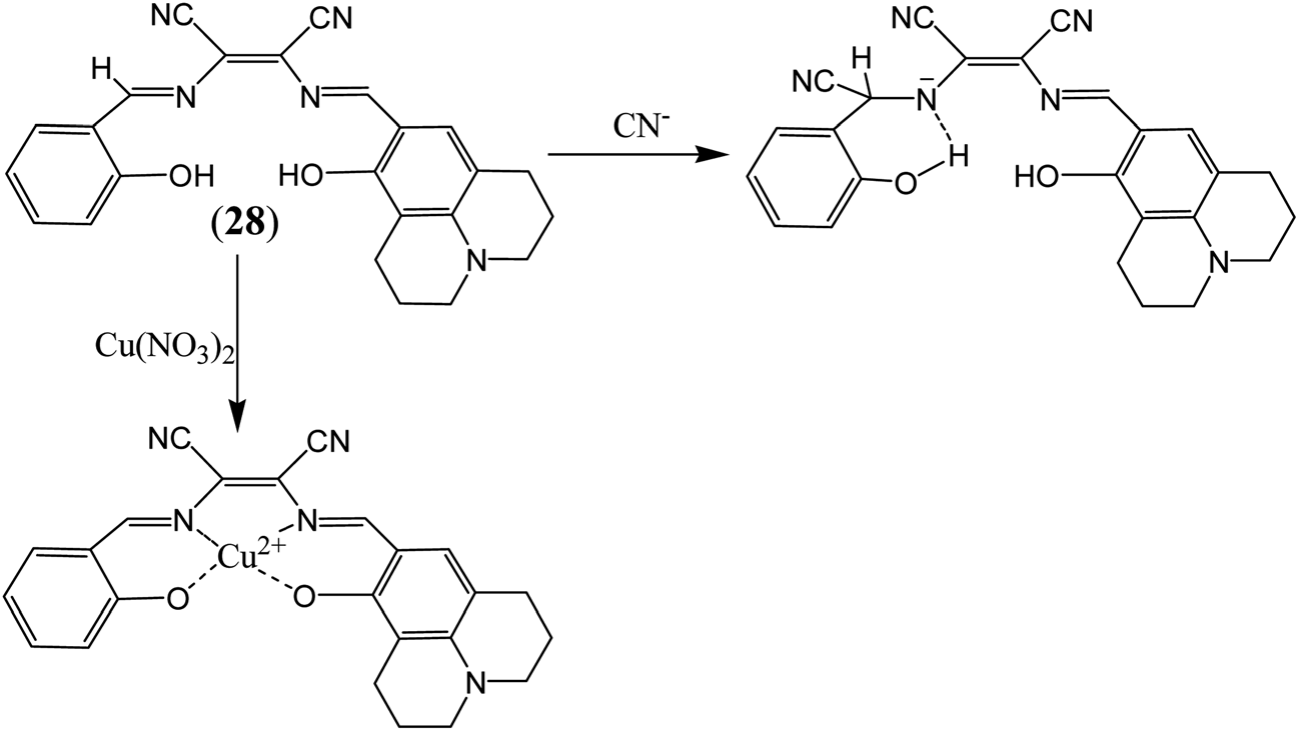
The sensing mechanism for sensor (28) for Cu^2+^ and CN^−^ ion.

More recently attention has been focused on the designing of multifunctional sensor molecules in which two or more binding sites are incorporated within a single framework that features simultaneous binding of multiple guest species. These multifunctional receptors are of inherent interest because of their potential applications in environmental protection, biological processes and industry.^58^ Several multifunctional sensor molecules using diaminomaleonitrile (DAMN) have been reported for sensing of multiple guest species. For instance, Kim and his group designed diaminomaleonitrile (DAMN) based colorimetric receptor for sensing of CN^−^ and Cu^2+^ ions.^59^ Receptor 28 was synthesized by reaction of 2-amino-3-(((*E*)-(8-hydroxy-2,3,6,7-tetrahydro-1*H*,5*H*-pyrido[3,2,1-*ij*]quinolin-9-yl)methylene)amino)maleonitrile with salicylaldehyde. In initial studies, it was observed that violet color solution of 28 changed to pale yellow color upon addition of CN^−^ ions while interfering ions like F^−^, AcO^−^, Cl^−^, Br^−^, H_2_PO_4_^−^, N_3_^−^ and SCN^−^ were remained unresponsive in DMSO/bis–tris buffer (9 : 1, v/v). UV-visible studies revealed that incremental addition of CN^−^ ion to 28 solution resulted disappearance of absorption peak at 560 nm with appearance of new peak at 445 nm along with isosbestic point at 375 nm. These observations indicated that addition of CN^−^ ion reduced the intramolecular charge transfer efficiency which lead to blue shift in absorption peak from 560 to 445 nm. Interestingly, ^1^H NMR studies showed that after addition CN^−^ ion, the CH=N proton signal shifted from 8.9 ppm to 5.7 ppm which indicated that CN^−^ ion acted as a nucleophile. In addition to this, cation sensing efficacy of 28 was examined by using nitrate salts of different metal ions in CH_3_CN. It was found that 28 showed instant color change from violet to pale yellow and distinct spectral change upon interaction with only Cu^2+^ ion. These changes were explained by the decrease in the push–pull effect of the ICT transition induced by interaction of Cu^2+^ ion to the imine groups and OH of receptor 28 (Fig. 14).

**Fig. 15:**
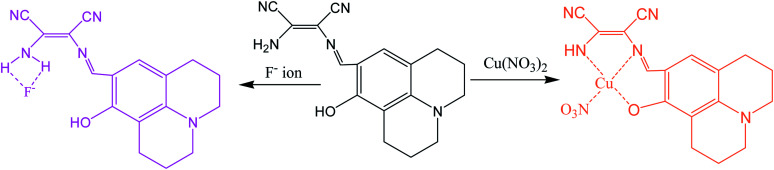
The sensing mechanism for sensor (29) for Cu^2+^ ion and F^−^ ion.

In an another report, a multifunctional chemosensor based on julolidine and diaminomaleonitrile moieties was designed for detection of both Cu^2+^ and F^−^ ion.^60^ The chemosensor (29) displayed absorption peak at 450 nm which was gradually decreased and simultaneously new peak at 375 nm was appeared with change in color from yellow to colorless on addition of Cu^2+^ ions. The sensing mechanism revealed the involvement of OH and NH_2_ groups of the receptor in Cu^2+^ ion binding led decrease intermolecular charge transfer transition (Fig. 15). It was found that sensor (29) can sense Cu^2+^ ion in much lower concentration than recommended by World Health Organization (WHO) in drinking water (30 mM) over a wide pH range of 4 to 12. Moreover, sensor (29) was also examined for sensing of different anions in similar condition because receptor having both phenolic and imine groups which can sense anions through hydrogen bonding. It was observed that upon addition of 600 equivalents of F^−^ ion, 29 showed significant spectral changes consistent with color changes from yellow to orange while other anions exhibited no change in absorption spectrum.

**Fig. 16:**
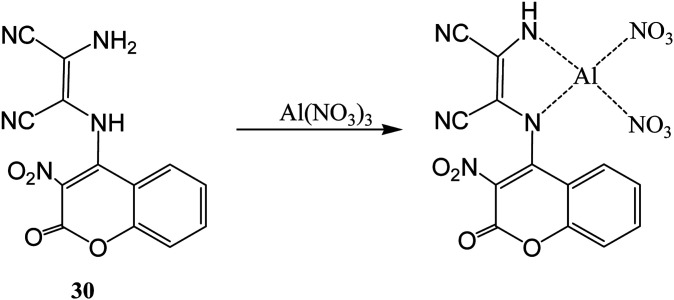
The sensing mechanism for sensor (30) for Al^3+^ ion.

A duel colorimetric sensor 2-(3-nitro-2-oxo-2*H*-chromen-4-ylamino)-3-aminomaleonitrile (30) was developed for the detection of Al^3+^ and F^−^ ions with detection limit of 38.2 μM for Al^3+^ ion.^61^ The sensor showed absorption peaks at 250, 334, and 432 nm which were decreased and a new peak at 302 nm was appeared on stepwise addition of Al^3+^ ion with significant colorimetric changes over other competitive ions like Ga^3+^ and In^3+^ in aqueous medium. The color changes might be attributed to binding of Al^3+^ ion with NH and NH_2_ groups of the receptor which result weakened ICT transition of sensor 30 (Fig. 16). Interestingly, it was found that sensor can be recycled and reused after treatment with ethylenediaminetetraacetic acid (EDTA). On the other hand, it was found that 30 showed selective colorimetric sensing towards F^−^ ion because of fluoride ion induced deprotonation process which led decrease in the intramolecular charge transfer.

A ninhydrin functionalized diaminomalenonitrile based sensor (31) was developed by condensation reaction between 2,3-diaminomalenonitrile and ninhydrin.^62^ Interestingly, sensor (31) exhibited blue color on simultaneous addition of Hg^2+^ and CH_3_COO^−^/F^−^ ions whereas addition of either Hg^2+^ or CH_3_COO^−^/F^−^ showed purple colour. These interesting switching behavior of sensor 31 was specific in terms of theses ion pairs as no other ion pairs exhibited similar behavior. These unique colorimetric behavior was further proven by UV-visible spectroscopy in which receptor 31 exhibited absorption peaks at 535 nm and 310 nm due to intramolecular charge transfer (ICT) band and sharp π–π transition band respectively. The addition of 2.0 equivalent of HgCl_2_ led no change in absorption spectrum as well as no visible color change. Interestingly, purple color of the receptor changed to blue as 2.0 equivalent of CH_3_COO^−^/F^−^ was added to the same solution. Along with this, in UV-vis spectrum of receptor, peak at 535 nm shifted to 590 nm while peak at 310 nm remained almost unaffected. Other anions like H_2_PO_4_^−^, Cl^−^, Br^−^, C_6_H_5_COO^−^ were not able to produce similar synergistic behavior with Hg^2+^ in terms of UV-vis spectral pattern or colorimetric responses.
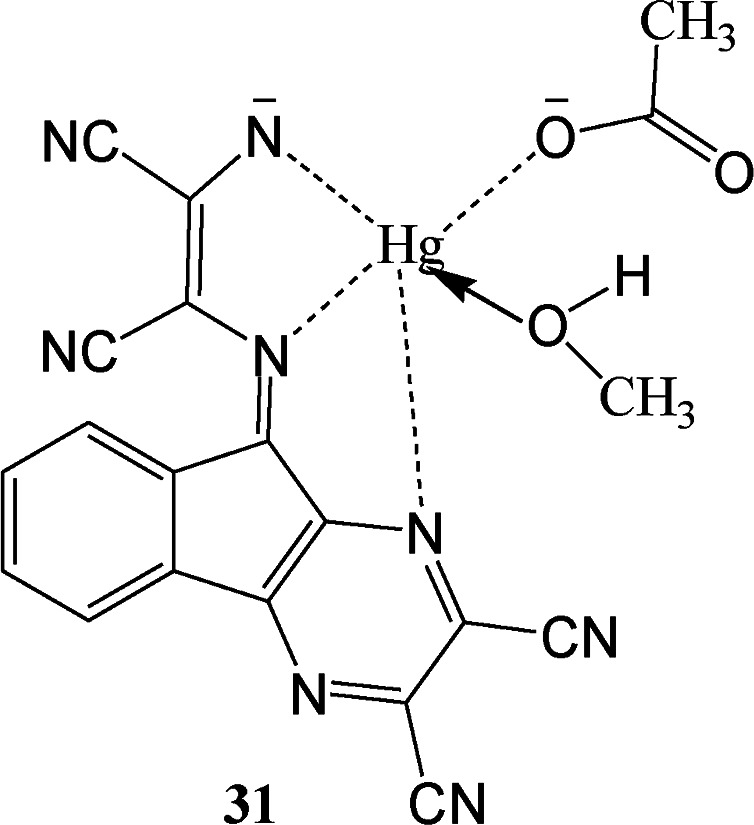


**Fig. 17:**
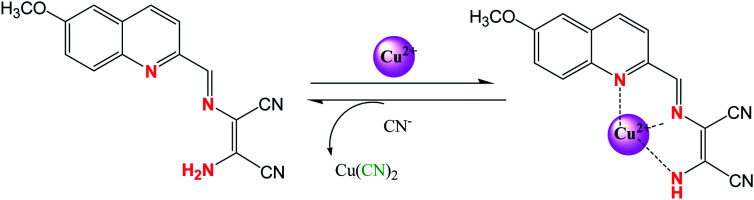
The sequential sensing mechanism for sensor (32) for Cu^2+^ an CN^−^ ion.

Some DAMN based chemosensor molecules were designed in which sensing of both metal and anion were carried out through metal ion displacement approach. For example, a quinoline-based chemosensor (32) was developed for sequential sensing of Cu^2+^ and CN^−^ ions.^63^ The sensor (32) exhibited remarkable colorimetric change with significant shift in absorption maxima from 390 nm to 537 nm on treatment with Cu^2+^ ion in aqueous solution. Therefore, sensor molecule was used for preparing test strips for monitoring of Cu^2+^ ion. In addition, 32–Cu^2+^ adduct was used for sequential monitoring of cyanide anion with dramatic colour change based on copper ion displacement mechanism as shown in Fig. 17.

**Fig. 18:**
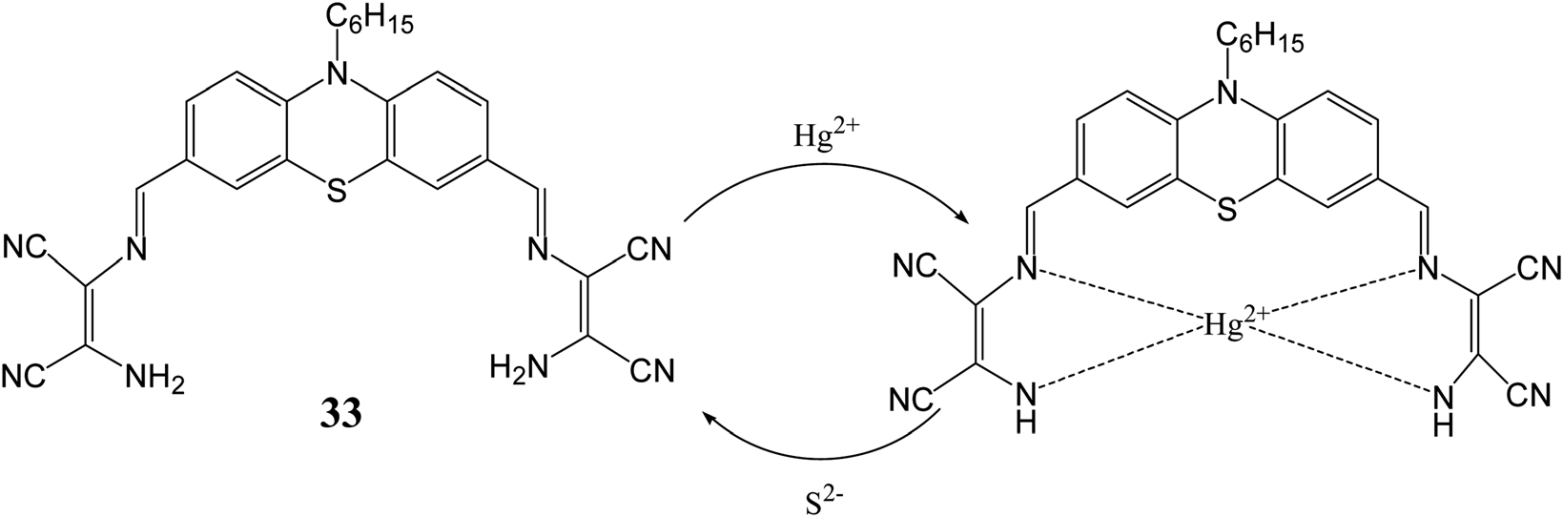
The sensing mechanism for sensor (33) for Hg^2+^ and S^2−^ ion.

By using similar approach, Singaravadivel *et al.* utilized phenothiazine moiety as fluorophore for synthesis of diaminomalenonitrile based chromo-fluorescent probe [3,3′-(((1*Z*,1′*Z*)-(10-hexyl-10*H*-phenothiazine-3,7-diyl)bis(methanylylidene))bis(azanylylidene))bis(2-aminomaleonitrile)] (33) for sensing of toxic Hg^2+^ and S^2−^ ions.^64^ Synthesized probe (33) exhibited emission peak at 575 nm upon excitation at 450 nm in ethanol/water solvent system. Addition of Hg^2+^ ion lead quenching of emission peak at 575 nm along with visible colorimetric change from yellow to brown whereas other ions such as Mn^2+^, Cu^2+^, Pb^2+^, Cd^2+^, Co^2+^, Zn^2+^, Fe^3+^, Ag^+^, Mg^2+^, Al^3+^, Cr^3+^, Ni^2+^, Na^+^ and K^+^, did not lead any change in the emission peak at 575 nm. This quenching behavior was ascribed by inhabitation of internal charge transfer process from phenothiazine moiety to DAMN unit after binding with Hg^2+^ ion. Further, it was observed that quenched fluorescence can be restored by adding 1.0 equivalent of Na_2_S solution. This is explained by the fact that mercury ions have high affinity for sulfur hence S^2−^ removed Hg^2+^ ion from 32–Hg^2+^ complex with regaining of emission peak at 575 nm (Fig. 18). It was also demonstrated that probe can be successfully used for selective imaging of Hg^2+^ and S^2−^ in living cells.

### DAMN based chemosensor for sensing of reactive oxygen species

**Fig. 19:**
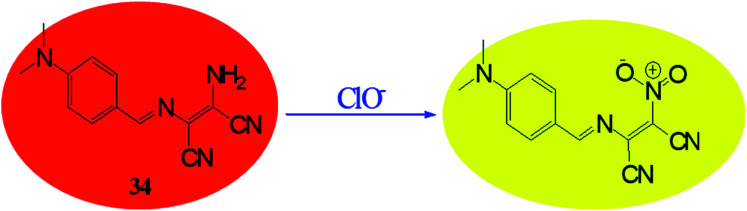
The formation of nitroso compound of sensor (34) on interaction with ClO^−^ ion.

Reactive oxygen species (ROS) have significant role in regulation of biological and pathological processes. Moderate level of ROS is crucial for normal cell functions.^65,66^ However, the overproduction of ROS increases oxidative stress, causing many detrimental effects on biological molecules such as proteins, phospholipids, RNA and DNA, which leads to inhibition of various protein functions and contributes to the progression of aging and numerous incurable human diseases.^67–72^ Various types of reactive oxygen species such as hydrogen peroxide (H_2_O_2_), superoxide (O_2_^−^), hypochlorous acid (HOCl), hydroxyl radical (HO˙), singlet oxygen and lipid hydro-peroxides are formed as natural by-product of the normal metabolism of oxygen. Among these ROS, hypochlorite (ClO^−^) is an important ROS, it is also extensively used in our daily life, such as a disinfectant for drinking water, household bleach *etc.* Hypochlorite is produced in living organisms by hydrogen peroxide and chloride ions through a chemical reaction catalyzed by the enzyme myeloperoxidase (MPO) and protect against the invasion of pathogens.^73–78^ Due to crucial role of hypochlorite various research groups are involved in developing molecular probes for easy and selective detection of hypochlorite ion. Since the colorimetric and fluorometric detections are easy and convenient than other analytical techniques, most efforts were put on developing colorimetric and fluorometric sensors. These sensors worked mainly on two approaches: (i) oxidation of functional group attached with signalling unit of sensor; (ii) cleavage of linkage between donor and acceptor group. On the basis of these approaches, there are reports where DAMN based chemosensors are used to detect ClO^−^ ion. For example, a simple diaminomaleonitrile based probe (34) synthesized by condensation reaction of 4-(dimethylamino)benzaldehyde and diaminomaleonitrile for ratiometric detection of ClO^−^ ion.^79^ A sharp colour change from the canary yellow to red upon interaction with ClO^−^ ion was advantage of this probe (34) for sensing of ClO^−^ ion through naked eye. The fluorometric analysis of the probe was performed and it showed that emission peak at 521 nm was decreases and a new emission peak evolved at 635 nm with addition of ClO^−^ solution. The intensity ratio of these two peaks was used for ratiometric detection of ClO^−^ ion. Interestingly, when comparing mass spectrometric and ^1^H NMR data of sensor (34) solution and sensor in presence ClO^−^ ion solution revealed oxidation of amino group of DAMN which resulted in the formation of nitro compound as shown in Fig. 19.

**Scheme 1:**
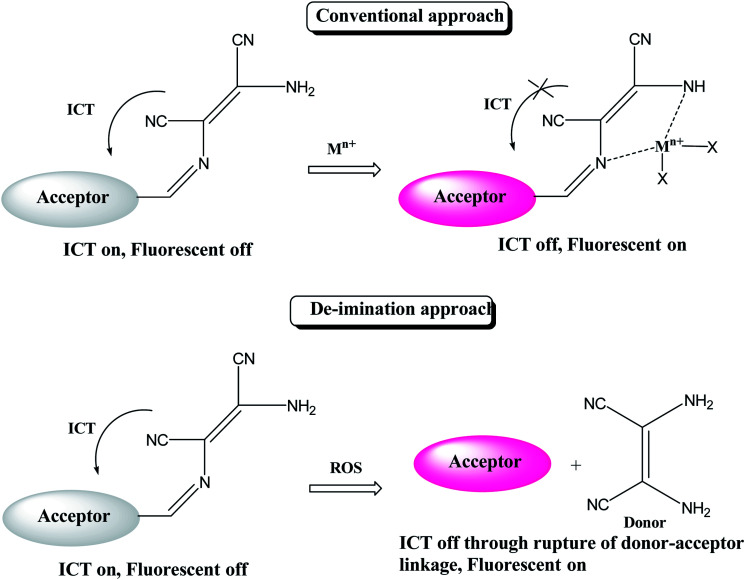
General approach for sensing of analytes by DAMN derived sensor molecules.

Generally, DAMN based sensors have been developed by conjugating of electron-withdrawing groups (acceptor) with electron-donating group (donor) *via* imine linkage. An intramolecular charge transfer (ICT) occurs from DAMN (donor) to acceptor upon excitation by light which make sensor non-fluorescent. The electron-donating DAMN binds with cation which inhibit intramolecular charge transfer (ICT) due to the loses of electron-donating ability of DAMN and make the sensor cation adduct fluorescent. In an another approach, the fluorescence intensity can be enhanced by breaking the imine linkage between DAMN and acceptor moiety by the specific analyte and the recovered parent acceptor moiety (fluorophore dye) rejuvenate its original intense emission peaks as depicted in Scheme 1.

**Scheme 2:**
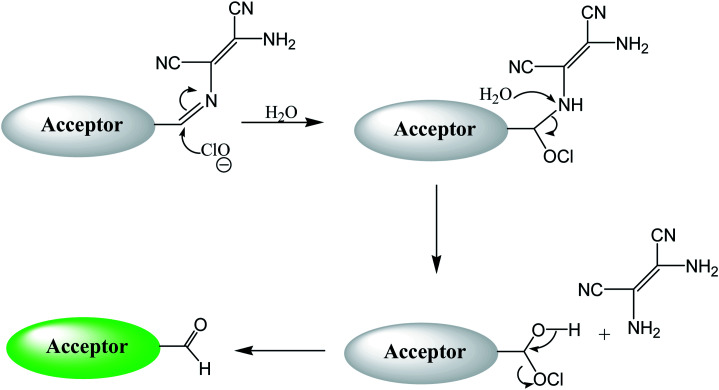
Sensing mechanism of hypochlorite by molecular probe.

By using virtue of this approach, different research groups have developed interesting sensor molecules for detection of hypochlorite ion by conjugating DAMN (donor) with different acceptors (fluorophores) like benzoxazole, carbazole, quinolone, phenanthroline, naphthalenone, pyrene, carbazole, coumarin derivatives, xanthene dyes and phenanthroimidazole moiety as shown in Table 1. Analysis of LOD values of sensors reported in Table 1 indicated that most of these sensors can detect ClO^−^ in the range of 10^−6^ to 10^−8^ M and DAMN attached to extended conjugated system exhibited lower detection limit. It was also observed that most of these sensor molecules exhibited low cytotoxic effect on cells thus can be used in imaging of living cells. In most cases the mechanism involves the attachment of ClO^−^ ion to the carbon atom of imine group followed by nucleophilic attack of H_2_O to form corresponding aldehyde and DAMN as shown in Scheme 2. The sensing mechanism was confirmed by NMR titration experiments, mass spectrum analysis and DFT calculations by most of research groups.^80,81,87^

**Table 1:** Comparison of different molecular probe for the detection of ClO^−^

Entry	Sensor	Detection limit	References
1	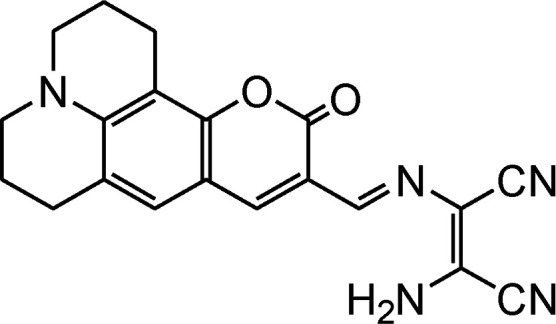	2.00 × 10^−4^ M	Yuan *et al.*^80^
2	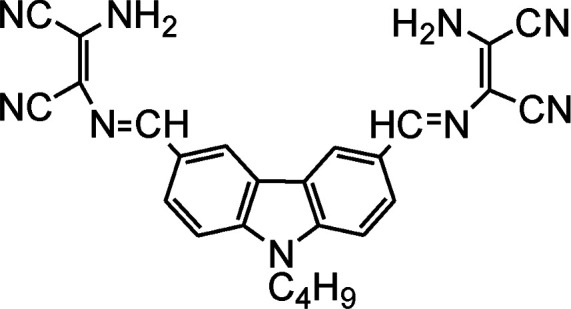	1.07 × 10^−6^ M	Goswami *et al.*^81^
3	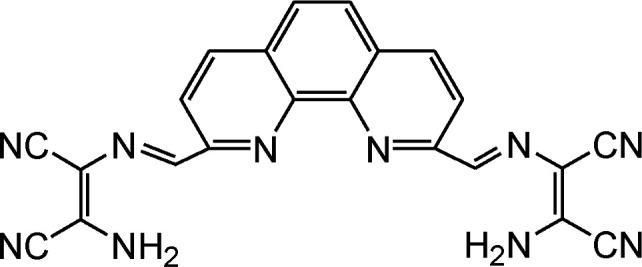	3.30 × 10^−6^ M	Goswami *et al.*^82^
4	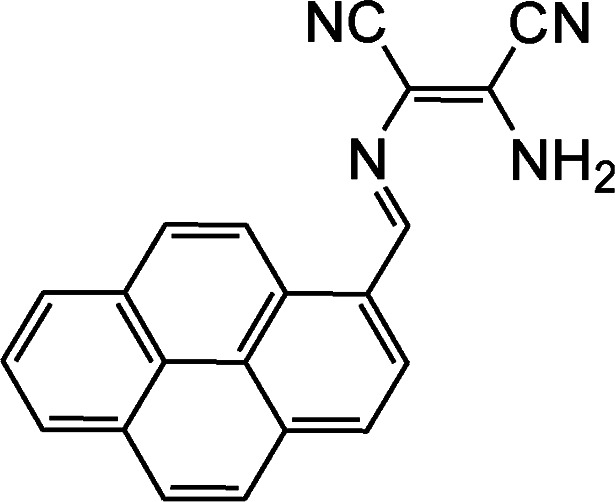	2.83 × 10^−6^ M	Yang *et al.*^83^
5	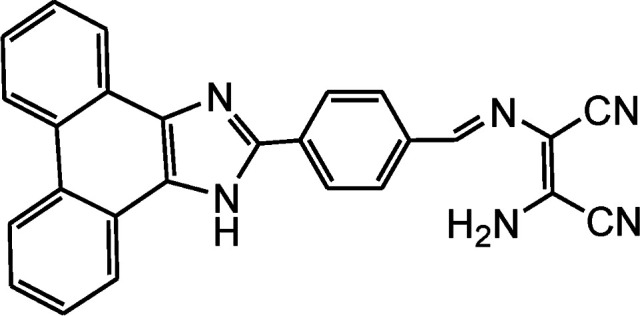	1.40 × 10^−8^ M	Zhao *et al.*^84^
6	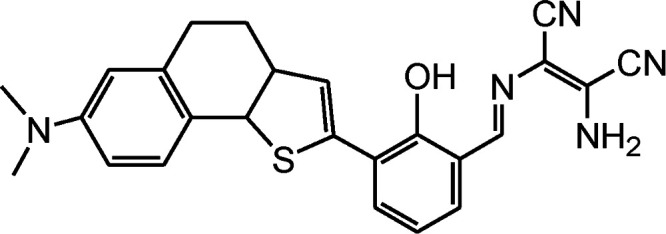	1.50 × 10^−7^ M	Ning *et al.*^85^
7	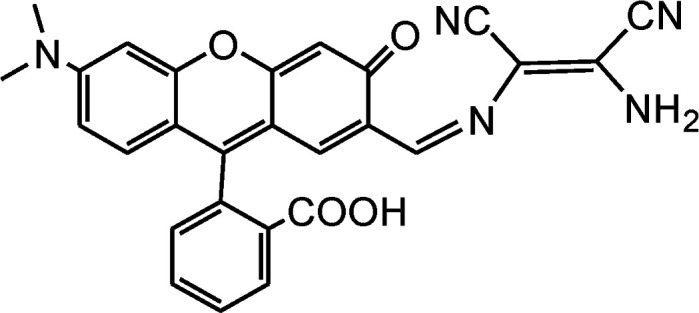	2.88 × 10^−8^ M	Zhang *et al.*^86^
8	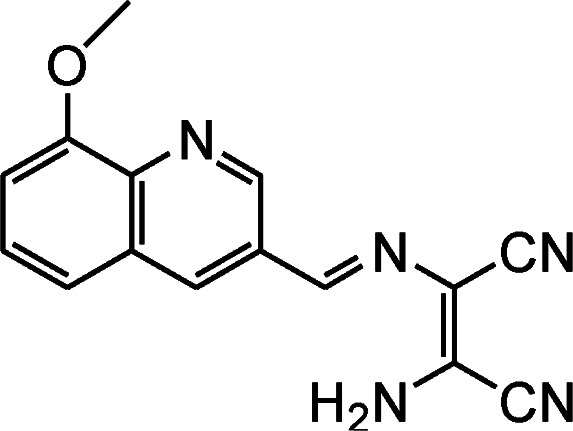	7.87 × 10^−7^ M	Das *et al.*^87^
9	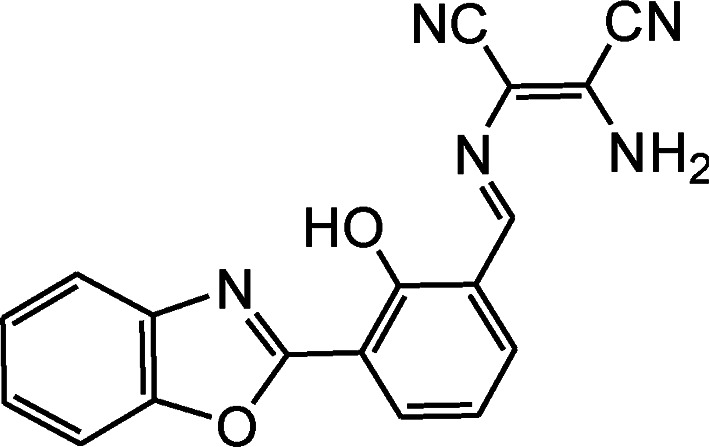	8.00 × 10^−8^ M	Chen *et al.*^88^
10	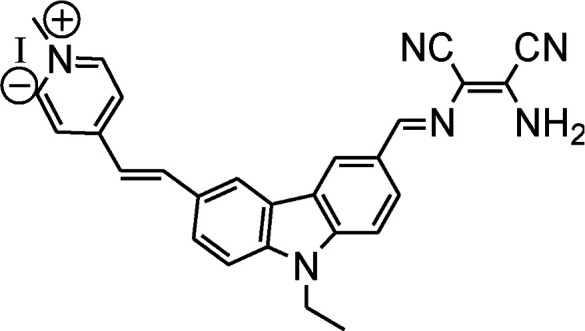	—	Feng *et al.*^89^

## Conclusion

Many approaches have been evolved to design effective molecular sensors capable of selective and sensitive sensing of cationic, anionic and neutral guest species with significant optical response. Many molecular structures with chromo sensing ability have been reported consisting of signal generating and binding sites. In these chemosensors, optical reporter is simply conjugated either directly or *via* short covalent spacer to binding sites which upon interaction with guest species produces changes in photophysical properties of the optical reporters. This review demonstrates how the field of DAMN based chemosensor research have flourished over a period of last 25 years. We have attempted to summarise research articles highlighting the use of 2,3-diaminomalenonitrile (DAMN) in the field of optical and colorimetric sensor for ionic and reactive oxygen species in this review. It is clear from this review that DAMN derived sensor molecules have ability to sense cations, anion and reactive oxygen species selectively in aqueous and other competitive media with detection limit of 10^−6^ to 10^−8^ M range. Further, availability of vast number of chromophore and fluorophore molecules can be employed to generate new sensors for efficient detection of targeted analytes. We hope that area of DAMN based sensors research will continue to thrive.

## Conflicts of interest

There are no conflicts to declare.

## Supplementary Material
